# Anti-inflammatory function of arctiin by inhibiting COX-2 expression via NF-κB pathways

**DOI:** 10.1186/1476-9255-8-16

**Published:** 2011-07-07

**Authors:** Sungwon Lee, Seulmee Shin, Hyunyul Kim, Shinha Han, Kwanghee Kim, Jeunghak Kwon, Jin-Hwan Kwak, Chong-Kil Lee, Nam-Joo Ha, Dongsool Yim, Kyungjae Kim

**Affiliations:** 1 College of Pharmacy, SahmYook University, Seoul 139-742, Republic of Korea; 2 School of Life Sciences, Handong Global University, Pohang 791-708, Republic of Korea; 3 College of Pharmacy, Chungbuk National University, Cheongju 361-763, Republic of Korea

## Abstract

**Background:**

Arctiin, isolated from *Forsythia suspensa *has been reported to have anti-inflammatory, anti-oxidant, antibacterial, and antiviral effects *in vitro*. However, there has been a lack of studies regarding its effects on immunological activity. The aim of this study is to investigate the anti-inflammatory potential and possible mechanisms of arctiin in LPS-induced macrophages.

**Methods:**

We investigated the mRNA and protein levels of proinflammatory cytokines through RT-PCR and western blot analysis, followed by a FACS analysis for surface molecule changes.

**Results:**

Arctiin dose dependently decreased the production of NO and proinflammatory cytokines such as IL-1β, IL-6, TNF-α, and PGE_2_, and it reduced the gene and protein levels as determined by RT-PCR and western blot analysis, respectively. The expression of co-stimulatory molecules such as B7-1 and B7-2 were also inhibited by arctiin. Furthermore, the activation of the nuclear transcription factor, NF-κB in macrophages was inhibited by arctiin.

**Conclusion:**

Taken together these results provide evidence of the bioactivity of arctiin in inflammatory diseases and suggest that arctiin may exert anti-inflammatory effect by inhibiting the pro-inflammatory mediators through the inactivation of NF-kB.

## Background

Non-steroidal anti-inflammatory drugs (NSAIDs) have been widely used in the treatment of acute and chronic inflammatory diseases, which play their therapeutic effects via inhibiting cyclooxygenase (COX) to prevent the production of pro-inflammatory prostaglandins. However, their long-term use shows the major side-effects of gastrointestinal diseases. Thus researchers have tried to screen new biological components from various plant sources including medicinal plants which inhibited COX with lower toxicity and higher anti-inflammatory activity on the great deal in the therapeutic application [1].

**Figure 1:**
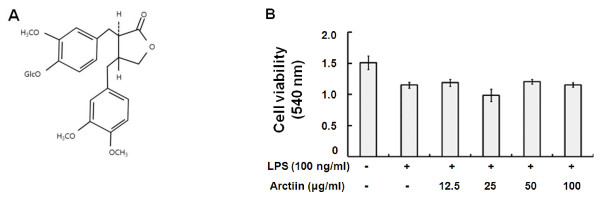
**Structure of arctiin (A); Cell viability (B).** Cell viability was evaluated as described in 'Materials and Methods'.

The fruit of *Forsythia suspensa Vahl, Forsythiae Fructus*, has been widely used in traditional medicines to treat swelling, gonorrhea, urination, hemorrhoids, tubercle, and other afflictions [2]. Arctiin is a lignan compound isolated from *Forsythiae Fructus *(Figure 1A); it has been found to significantly induce cell detachment and decrease the number of PC-3 cells in human prostate cancer [3]. Moreover, it has been demonstrated to possess many kinds of bioactivities [4] and a number of important pharmacological properties including being demutagenic [5,6] cytotoxic, anti-proliferative [7,8], platelet activating factor (PAF) antagonistic [9], calcium antagonistic [10], and anti-carcinogenetic. In animal studies, arctiin effectively inhibited the formation of 12-O-tetradecanoylphorbol-13-acetate (TPA)-induced ear edema in mice [11]. However, there has been a lack of studies regarding the effects of arctiin on inflammation.

Chronic inflammation and infection has been demonstrated to lead to an upregulation of a series of enzymes and signaling proteins in affected tissues and cells. Inflammation has been shown to be a multi-step process, mediated by activated inflammatory and immune cells including macrophages and monocytes [12]. Inflammatory reactions, phagocytosis, natural cytotoxicity, cytokine production, antibody response, and cellular immunity are defensive mechanisms that have been suggested to be modulated by therapeutic doses of antimicrobial agents [13]. Activated macrophages include the inducible forms of nitric oxide synthase (iNOS) and cyclooxygenase-2 (COX-2), which have been reported to be responsible for increasing the levels of nitric oxide (NO) and prostaglandins (PGs), the overproduction of proinflammatory cytokines (e.g. TNF-α, IL-6, IL-1β) and inflammatory mediators (e.g. PGE_2_, NO), and the mediation of many inflammatory diseases [14].

Costimulatory molecules are one class of receptors, which have recently been implicated as fulfilling this role in the innate immune response. B7-1 and -2 represent one class of costimulatory receptors. They consist of structurally related, cell-surface protein ligands, which bind to receptors on lymphocytes that regulate immune responses. In addition, they are stimulated via CD28 while CTLA4 serves as both a stoichiometric inhibitor of CD28-B7-1/-2 engagement as well as serving to directly induce immunosuppressive signals within dendritic cells [15].

In this study, we evaluated the potential of arctiin as a therapeutic modality for inflammation in RAW264.7 mouse macrophage cells as well as in primary peritoneal macrophages. Our results demonstrated that arctiin largely inhibited the excessive production of inflammatory mediators such as NO, PGE_2_, TNF-α, IL-1β and IL-6 as well as the suppression of COX-2 through the inhibition of NF-kB translocation pathway.

## Methods

### Chemicals and reagents

Dulbecco's Modified Eagle Medium (DMEM)-1640, fetal bovine serum (FBS), and penicillin/streptomycin were purchased from Hyclone (Logan, USA). *Escherichia coli *lipopolysaccharide (LPS) was purchased from sigma (St. Louis, USA). β-actin, i-NOS, COX-2, p65, p-IκBα, PE-B7-1, and FITC-B7-2 anti-bodies were purchased from BD Pharmingen™ (San Jose, USA). Enzyme immunoassay kits for the measurement of PGE_2_, IL-1β, IL-6, and TNF-α were purchased from R&D system (Minneapolis, USA).

### Isolation of arctiin from Forsythiae Fructus

*Forsythiae Fructus *obtained from an herbal market in Seoul, Korea, was extracted three times with hot MeOH (3 hours) and then evaporated at 40°C under reduced pressure to dryness. The MeOH extract was then resuspended in distilled water and successively partitioned with CHCl_3_, EtOAc, and n-BuOH. The BuOH fraction was then loaded onto a silica gel column and eluted with MeOH-CHCl_3 _mixtures (1:5 to 1:1). The result was white amorphous powders, which were identified as authentic samples using spectrometric data of nuclear magnetic resonance (^1^H-NMR), mass spectrometry (MS). ^1^H-NMR (300 MHz), DMSO-d_6 _: δ 6.97(1H, d, *J *= 8.3, H-5), 6.82 (1H, d, J = 8.0, H-5'), 6.77 (1H, d, *J *= 1.7, H-2), 6.65 (1H, dd, *J *= 8.3, 1.7, H-6), 6.65 (1H, s, H-2'), 6.59 (1H, dd, *J *= 8.0, 1.8, H-6'), 4.83 (1H, d, *J *= 7.3, H-1''), 4.00 (2H, m, H-9'), 3.70 (3H, s, OCH_3_), 3.69 (6H, s, OCH_3_), 2.76 (2H, m, H-7'), 2.54 (4H, m, H-7, 8, 8'). Positive FABMS (*m/z*): 557 (M + Na^+^), 372 (M + gluco), 154, 136. The white amorphous powder compound analyzed by NMR and MS was identified to arctiin (Figure 1A).

### Animals

ICR mice (6-8 weeks old, specific pathogen-free) were obtained from Orient-Bio Co. (Seongnam, Korea). Animals were fed with standard laboratory chow (PMI Lab Diet, Richmond, USA) and autoclaved distilled water (DW). They were acclimatized in an animal facility (Sahmyook University, Korea) and maintained at 22 ± 2°C in 50 ± 10% relative humidity and a light/dark (12 hrs/12 hrs) cycle for at least 7 days prior to the experiments.

### Cell culture

Male ICR mice (6-8 weeks) were intraperitoneally injected with 1.5 ml thioglycollate broth for recruitment of macrophages. RAW 264.7 cells were obtained from the American Type Culture Collection (ATCC, Rockville, USA). These cells were grown at 37°C in DMEM medium supplemented with 10% FBS and 1% (v/v) penicillin (10,000 U/ml)/streptomycin (10,000 U/ml) in a humidified 5% CO_2_-95% air incubator under standard conditions.

### Cell viability assay

A commercially available cell viability assay was employed to evaluate the cytotoxic effect of arctiin using thiazolyl blue tetrazolium bromide (SIGMA, USA). RAW264.7 cells (2 × 10^5 ^cells/well) were plated with various concentrations of arctiin in 96-well microtiter plates (Nunc, Roskilde, Denmark) and were then cultured at 37°C in a 5% CO_2 _incubator. Subsequently, 50 μl of MTT solution was added to each well, and the cells were then cultured for 4 hrs at 37°C in the same incubator. Following this, 100 μl of solubilized solution were added to each well and the plate was allowed to stand overnight in the incubator. The optical density (OD) was then measured at 560 nm by a microplate reader (Molecular devices, USA).

### Nitrite measurement

RAW 264.7 cells were added to each well (200 μl; 1 × 10^6 ^cells/ml) of a flat-bottomed 96-well plate according to the following treatment condition: LPS (100 ng/ml), LPS/arctiin (12.5, 25, 50, 100 μg/ml), and media only (DMEM-10). Nitric oxide was measured in culture supernatants by reaction with the Griess reagent (1% sulfanilamide and 0.1% N-[1-naphthy]-ethylenediamine dihydrochloride in 5% phosphoric acid; Roche) to 100 μl of culture supernatant for 15 min at room temperature in the dark. The absorbance at 540 nm was then determined using a microplate reader (Molecular devices, USA) and a standard curve was generated using NaNO_2_.

### Determination of pro-inflammatory cytokines and PGE_2_

RAW 264.7 cells and primary macrophages were cultured in 12-well flat plates at a density of 5 × 10^6 ^cells/well. The cells were then treated with various concentrations of arctiin and subsequently stimulated with LPS (100 ng/ml) at 37°C for 48 hrs in humidified air with 5% CO_2_. The supernatants were then collected and measured for TNF-α, IL-1β, IL-6, and PGE_2 _by an enzyme-linked immunosorbent assay (ELISA) according to the manufacturer's protocol.

### RT-PCR (reverse transcription polymerase chain reaction)

Total RNA was extracted from macrophages using the RNeasy Mini Kit (QIAGEN, USA) in an RNase-free environment. The reverse transcription of 1 μg RNA was carried out using M-MLV reverse transcriptase (Promega, USA), oligo (dT) 16 primer, dNTP (0.5 mM) and 1 U RNase inhibitor. After incubation at 65°C for 5 min and 37°C for 60 min, M-MLV reverse transcriptase was inactivated by heating at 70°C for 15 min. The polymerase chain reaction (PCR) was performed in 50 mM KCl, 10 mM Tris-HCl (pH8.3), 1.5 mM MgCl_2_, and 2.5 mM dNTPs with 5 units of Taq DNA polymerase and 10 pM of each primer set for IL-1β, IL-6, TNF-α, iNOS, and COX-2. The cDNA was amplified by 35 cycles of denaturing at 94°C for 45 s, annealing at 60°C for 45 s, and extension at 72°C for 1 min. Final extension was performed at 72°C for 5 min. The PCR products were electrophoresed on 1.5% agarose gels and stained with ethidium bromide. The primer sequences were as follows: 5'- AGC TCC TCC CAG GAC CAC AC-3' (forward), 5'-ACG CTG AGT ACC TCA TTG GC-3' (reverse) for i-NOS, 5'-AAG AAG AAA GTT CAT TCC TGA TCC C-3' (forward), 5'-TGA CTG TGG GAG GAT ACA TCT CTC-3' (reverse) for COX-2, and 5'-GTG GGC CGC CCT AGG ACC AG-3' (forward), 5'- GGA GGA AGA GGA TGC GGC AG T-3' (reverse) for β-actin as a control for PCR. The band intensity was quantified by densitometric analysis (Infinity 3026, Vilber Lourmat, France).

### Preparation of cytosolic and nuclear extracts

The cells were collected after culture and washed twice with cold PBS, resuspended in hypotonic buffer (10 mM HEPES, pH 7.9, 10 mM KCl, 1.5 mM MgCl_2_, 0.2 mM PMSF, 0.5 mM DTT, 10 μg/ml aportinin). After 15 min incubation on ice, the cells were lysed by the addition of 0.1% NP-40 and vigorous vortexing for 1 min. The nuclei were pelleted by centrifugation at 12,000 × g for 1 min at 4°C and resuspended in high salt buffer (20 mM HEPES, pH 7.9, 25% glycerol, 400 mM KCl, 1.5 mM MgCl_2_, 0.2 mM EDTA, 0.5 mM DTT, 1 mM NaF, 1 mM sodium orthovanadate). The cytosolic and nuclear extracts were stored in aliquots at -70°C.

### Western blot analysis

RAW264.7 cells were washed with phosphate-buffered saline (PBS) and lysed using lysis buffer (1% SDS, 1.0 mM sodium vanadate, 10 mM Tris-Cl buffer, pH 7.4) for 5 min. Further, 20 μg of protein from the cell lysates were applied to 8-12% SDS-polyacrylamide gels and then transferred to nitrocellulose membranes. The membranes were blocked in 5% skim milk solution for 1 h. They were then incubated with anti-TNF-α, anti-IL-1β, anti-IL-6, anti-iNOS, anti-COX-2, anti-p-IκBα, or anti-p65 monoclonal antibodies for 2 h and subsequently washed 3 times with PBS. After incubation with an AP-labeled secondary antibody for 2 h, the bands were visualized using an alkaline phosphatase substrate (VECTOR, USA).

### Flow cytometry

RAW 264.7 cells (1 X 10^6 ^cells/ ml) were cultured in Petri-dishes. The cells were treated with various concentrations (12.5, 25, 50, 100 μg/ ml) of arctiin in the presence of LPS (100 ng/ ml). The dishes were incubated at 37°C for overnight in humidified 5% CO_2 _incubator under standard conditions. The cells were then washed with PBS. The washed cells were blocked with staining buffer containing 10% normal mouse serum (NMS) for 20 min on ice. The blocked cells were incubated with co-stimulatory molecules such as B7-1 and B7-2 antibody (BD Biosciences, San Jose, USA) for 20 min on ice. The incubated cells were washed three times with staining buffer and then fixed by 1% paraformaldehyde in PBS. The fixed cells were measured by flow cytometry (Beckman coulter, Brea, USA).

### Statistical analysis

All data are presented as mean ± SEM values. Significant differences (*P < 0.05*) between groups were evaluated using a one-way analysis of variance with SPSS (Chicago, IL, USA) for Windows and Duncan's Multiple Range Test where appropriate.

## Results

Prior to evaluating whether arctiin showed anti-inflammatory activity, we examined its effect on cell growth in RAW264.7 cells and found that arctiin did not affect normal cell growth at concentrations up to 100 μg/ml (Figure 1B). Thus, in the following experiments, arctiin was studied at concentrations up to 100 μg/ml in order to exclude any effects on the normal growth status of cells.

### Effect of arctiin on PGE_2_ and NO production in macrophages

**Figure 2:**
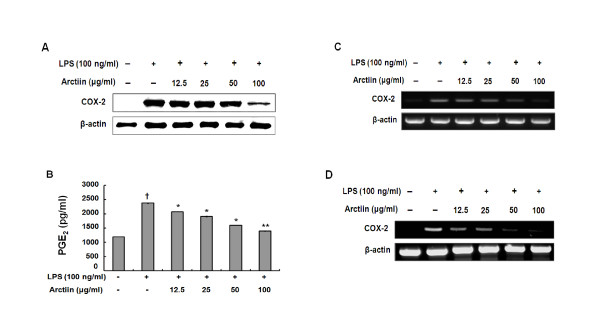
**Arctiin inhibits the production of COX-2 (A), PGE_2 _(B) and mRNA (C, D) in LPS-stimulated macrophages**. RAW 264.7 cells (A-C) and primary macrophages (D) were treated with different concentrations of arctiin (12.5~100 μg/ml) in the presence of LPS (100 ng/ml) and was monitored as described in 'Materials and Methods'. Each bar represents the means ± S.D. from three separate experiments. *^††^P*< 0.01 compares to the control. *P< 0.05, **P< 0.01 compared to the LPS.

In the present study, we examined whether or not arctiin suppress macrophages activation induced by LPS, one of the most potent macrophages activation factors. Arctiin significantly suppressed COX-2 protein expression (Figure 2A) in LPS-stimulated RAW 264.7 cells. The pro-inflammatory mediator, PGE_2 _is also generated by the COX-2 enzyme in response to stimulation by LPS. Results showed that arctiin significantly inhibited PGE_2 _production (Figure 2B) and mRNA of COX-2 in RAW 264.7 cells (Figure 2C) and primary macrophages (Figure 2D) by western blot analyses and subsequent RT-PCR.

**Figure 3:**
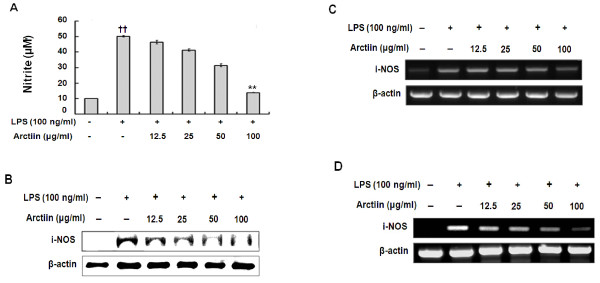
**Arctiin inhibits the production of NO (A), expression of iNOS protein (B), and mRNA (C, D) in LPS-stimulated macrophages**. RAW 264.7 cells (A-C) and primary macrophages (D) were treated with different concentrations of arctiin (12.5 ~ 100 μg/ml) in the presence of LPS (100 ng/ml) and was monitored as described in 'Materials and Methods'. Each bar represents the means ± S.D. from three separate experiments. *^††^P*< 0.01 compares to the control. **P< 0.01 compared to the LPS.

NO is known to be a pro-inflammatory mediator in inflammatory diseases. Several studies have demonstrated that overproduction of NO by iNOS was associated with inflammatory responses and also with serious disorders, including RA. Therefore, we investigated whether arctiin inhibits NO production in macrophages that were activated with LPS. Interestingly, in LPS (100 ng/ml) stimulated RAW264.7 cells, when various concentrations of arctiin (12.5, 25, 50, 100 uGu/ml) were added to the culture media at the time of cell stimulation, LPS-induced production of NO was significantly inhibited in a dose-dependent manner (Figure 3A). Subsequent RT-PCR and western blot analyses showed that arctiin inhibited protein expression of i-NOS (Figure 3B) and mRNA (Figure 3C) in RAW 264.7 cells as well as the primary macrophages (Figure 3D).

### Arctiin down-regulates production of pro-inflammatory cytokines

**Figure 4:**
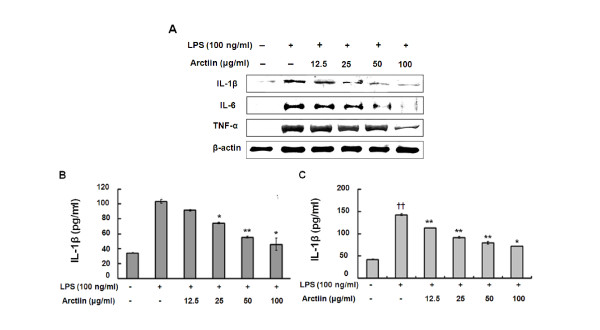
**Arctiin inhibits the production of pro-inflammatory cytokines in LPS-stimulated macrophages**. RAW 264.7 cells (A:Western blot, B, D, and F:ELISA) and primary macrophages (C, E, and G:ELISA) were treated with different concentrations of arctiin (12.5~100 μg/ml) in the presence of LPS (100 ng/ml) and was monitored as described in 'Materials and Methods'. Each bar represents the means ± S.D. from three separate experiments. *^†^P*< 0.05, *^††^P*< 0.01 compares to the control. *P< 0.05, **P< 0.01 compared to the LPS.

The *in vitro *anti-inflammatory activity of arctiin was monitored by evaluating the gene and protein expression levels of inflammation-related enzymes (iNOS and COX-2) and several proinflammatory cytokines (IL-1β, IL-6, and TNF-α) with ELISA and western blot analysis. As shown in Figure 4A, arctiin significantly suppressed the protein expression of pro-inflammatory cytokines in LPS-stimulated macrophages. Moreover, productions of cytokine were significantly attenuated by 100 μg/ml of arctiin in the both RAW 264.7 cells (Figure 4B, D, and F) and primary macrophages (Figure 4C, E, and D).

### Effect of arctiin on the expression of co-stimulatory molecules

**Figure 5:**
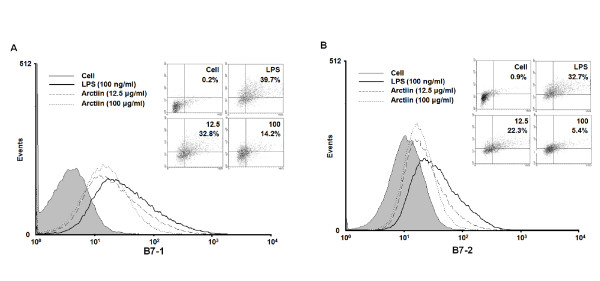
**Arctiin inhibits the expression of co-stimulatory molecules in LPS-stimulated macrophages**. RAW 264.7 cells (A-B) were treated with different concentrations of arctiin (12.5~100 μg/ml) in the presence of LPS (100 ng/ml) and was monitored as described in 'Materials and Methods'. The surface B7-1 (A) and B7-2 (B) molecules were labeled with either anti-B7-1, B7-2 and the cell were stained using anti-Vβ8.1+8.2-FITC, anti-Vβ2-PE, anti-Vβ2-FITC (shaded histogram), which served as an isotype control for the nonspecific binding.

Adhesion molecules play an important role in the macrophage activation process. RAW264.7 cell surface expression of B7-1 and B7-2 was examined using flow cytometry. Results demonstrated that arctiin inhibited cell surface molecules in a dose-dependent manner. Further, LPS-stimulated RAW264.7 cells treated with a high concentration of arctiin (100 μg/ml) had a greater reduction than other concentrations (Figure 5).

### Effect of arctiin on the activation of NF-κB

**Figure 6:**
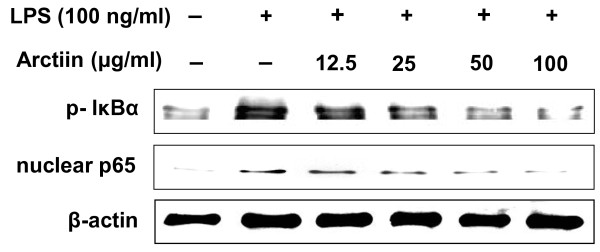
**Arctiin inhibits the IκBα phosphorylation in cytoplasm and nuclear translocation of NF-κB p65 in LPS-stimulated macrophages**. RAW 264.7 cells were treated with different concentrations of arctiin (12.5~100 μg/ml) in the presence of LPS (100 ng/ml) and was monitored as described in 'Materials and Methods'.

NF-кB proteins are present in the cytoplasm as inactive heterodimers composed of two subunits, P50 and P65, and are bound to the inhibitory protein IκBα which prevents it from the translocation into the nucleus of the cell [16]. Upon stimulation, IκBα is phosphorylated and proteolytically degraded through a 26S proteasome-mediated pathway which facilitates NF-кB translocation into the nucleus and regulates gene transcription [17]. To investigate whether arctiin could affect nuclear translocation of NF-кB, western blot analysis of NF-кB p65 was carried out using RAW264.7 cell lysates. The amount of NF-кB p65 was markedly increased upon exposure to LPS alone, whereas arctiin inhibited it (Figure 6). Furthermore, we examined how arctiin modulated translocation of NF-κB, western blot analysis of phosphorylation of I-κBα in cytoplasm. Arctiin significantly attenuated IκBα phosphorylation in RAW 264.7 cells (Figure 6). These results suggest that inhibition of i-NOS, IL-1β, IL-6, TNF-α, and COX-2 gene expression by arctiin may have been due to the down regulation of NF-κB activation.

## Discussion

Arctiin is lignin compound isolated from *Forsythiae Fructus*. The anti-cancer [3,7,8] and platelet activating factor antagonistic effects [9] of arctiin have been well documented but the mechanisms underlying anti inflammatory effect are still not understood. The present study found that arctiin significantly inhibited the effects of LPS by suppressing a key inflammatory pathway related to NF-κB, PGE_2 _and NO production, and pro-inflammatory cytokines expression.

Inflammation is a reaction of the body to injury or to infectious, allergic, or chemical irritation. Leukocytes destroy harmful microorganisms and dead cells, preventing the spread of the irritation and permitting the injured tissue to repair itself. However, excessive or persistent inflammation causes a variety of pathological conditions, such as bacterial septic shock, and rheumatoid arthritis [18,19]. Inflammatory mediators (e.g. nitric oxide and pro-inflammatory cytokines) have been demonstrated to be critically involved in the development of inflammatory diseases.

Macrophages play important roles in regulating cell-mediated immune responses, such as adaptive immune, innate immune, and allergic reactions, as well as inflammation in response to microbes and microbial products such as LPS [20]. In addition to the well-known function of endocytosis, macrophages can be induced to secrete nitric oxide and a series of cytokines including TNF-α, IL-1β, IL-6, and IL-12 that express NF-κB-dependent i-NOS [21,22] and COX-2 [23]. Many diseases, such as arteriosclerosis, chronic hepatitis and pulmonary fibrosis, involve the overproduction of inflammatory mediators [24-27] and thus, inhibiting their production may serve to prevent or suppress a variety of inflammatory diseases, including rheumatoid arthritis (RA), sepsis, and endotoxemia. Despite the exact cause of autoimmune disease remaining obscure, deregulated overproduction of pro-inflammatory cytokines and a disruption in the regulation of cytokine signal transduction have been indicated as underlying mechanisms of some autoimmune diseases such as RA and Crohn's disease [28,29].

Nitric oxide is synthesized via the oxidation of arginine by a family of NOS, and it plays a vital role in regulating physiological processes, such as blood vessel tone and neurotransmission, as well as in host defense and immunity [30,31]. Pro-inflammatory cytokines, IL-1β, IL-6, and TNF-α, have attracted more attention in that they can be localized to the infected tissue, manifested systemically throughout the body, and cause vasodilation as well as symptoms of inflammation [32-34]. Our findings that arctiin inhibits the formation of NO and pro-inflammatory cytokines showed the importance of arctiin as an anti-inflammatory compound. The reduced NO production by arctiin was a consequence of an inhibition of iNOS, the key enzyme responsible for NO production under pathological conditions.

PGE_2 _plays major roles in the angiogenesis of synovium through the expression of vascular endothelial growth factors [35], synovial inflammation, and joint erosion in RA [36]. Further, in the prostaglandin biosynthesis pathway, COX-2 is the key enzyme that catalyzes the conversion of arachidonic acid to PGE_2_. It is generally accepted that PGE_2 _is produced by COX-2 at sites of inflammation, and that COX-1, another constitutive isoform, is relevant in the production of prostaglandins that regulate normal cellular processes such as vascular homeostasis regulation, gastric mucosal protection and renal integrity maintenance [37]. In the present study, we found that arctiin suppressed PGE_2 _production via inhibition of COX-2 enzyme activity and this may in part be responsible for some of anti-inflammatory properties of this compound.

The transcriptional factor NF-κB is important for the expression of immune and inflammatory genes. The activated NF-κB then binds κB motifs in the promoters via its p65 subunit, leading to expression of several inflammatory genes. Because NF-κB transcription factors are uniquely positioned downstream of multiple innate and adaptive signaling pathways, they seem ideally placed to integrate and coordinate innate and adaptive signals required for formation of productive immune responses. In the present study, we found that arctiin could inhibit the NF-κB nucleus translocation induced by LPS through a reduction in IκB phosphorylation status.

The B7 family is related immunoglobulin supergene family members that are expressed by multiple cell types involved in antigen presentation. Both B7-1 and B7-2 are constitutively expressed on dendritic cells and are regulated on monocytes, macrophages, B cells, and T cells following activation. In agreement with our findings, arctiin has also been shown to suppress co-stimulatory molecules such as B7-1 and B7-2 in macrophages.

In summary, these results suggest that arctiin has anti-inflammatory effects on macrophages through the reduced pro-inflammatory cytokines are associated with NF-κB inactivation and the suppression of NF-kB-regulated proteins, and other bioactive substances as well as through inhibition of the expression of co-stimulatory molecules.

## Competing interests

The authors declare that they have no competing interests.

## Authors' contributions

SL participated in the design of this study and performed the statistical analysis in whole research. SS carried out the Western blot assay of iNOS, COX-2, and proinflammatory cytokines. HK carried out the RT-PCR assay. SH carried out the FACS analysis in B7 family. KK carried out the preparation of peritoneal macrophages, participated in the maintenance of SPF room and care of animal. JK carried out the cytokine assay (ELISA). JHK participated in the design of the study, and proofread a manuscript about *in vitro *experiments design. CKL participated in the design of the study, and proofread a manuscript about *in vivo *experiments design. NJH participated in the test of toxicity of this compound, and proofread a manuscript about cell toxicity. DY carried out the separation of arctiin from *Forsythiae Fructus*. KK conceived of the study, and participated in its design and coordination. All authors read and approved the final manuscript.
